# Oseltamivir use amongst hospitalized patients infected with influenza

**DOI:** 10.1111/irv.12260

**Published:** 2014-07-04

**Authors:** Michael Herman, Marek Smieja, Susan Carruthers, Mark Loeb

**Affiliations:** a Department of Medicine, McMaster UniversityHamilton, ON, Canada; b Department of Pathology and Molecular Medicine, McMaster UniversityHamilton, ON, Canada; c Department of Clinical Epidemiology and Biostatistics, McMaster UniversityHamilton, ON, Canada; d Institute for Infectious Diseases Research, McMaster UniversityHamilton, ON, Canada

**Keywords:** hospitalization, influenza, oseltamivir

To the editor

Influenza, together with pneumonia, is the leading infectious cause of death in Canada and the United States, especially in individuals with risk factors such as cardiac or pulmonary disorders, diabetes mellitus, cancer, immune suppression, renal disease, morbid obesity, chronic aspiration, patients over the age of 65, pregnant women and residents of nursing homes.^1^,^2^ Oseltamivir, the most effective and practical anti-influenza agent, is recommended in the treatment of all hospitalized patients; however, little is known about the compliance with this recommendation. Previous observational studies have shown a prescription rate of around one-third of high-risk patients.^3^,^4^ Recent observational data suggest that early use of oseltamivir is associated with reduction in mortality in hospitalized patients.^5^

The goal of this study was to assess prescription prevalence of oseltamivir amongst hospital inpatients with laboratory-confirmed influenza and to determine patient characteristics associated with antiviral prescription.

Data were collected on inpatients with laboratory-confirmed influenza infection between 2007 and 2009 in a tertiary care hospital in Hamilton, Canada. Age, comorbid conditions, antibiotic use, length of stay and oseltamivir use were recorded and used to determine the proportion of hospitalized patients receiving oseltamivir. Independent predictors of oseltamivir prescription, such as the presence of comorbidity, were determined using logistic regression analysis. All statistical analysis was performed using version 20 of IBM SPSS software (IBM Corp., Armonk, NY, USA).

A total of 384 patients were reviewed. Mean age (SD) was 64·5 (19·3) years; 55% were female; 86·2% were within the high-risk category; and antibiotics were prescribed to 74% of patients. Oseltamivir was prescribed in 19·5% of patients overall. Predictors found to be significantly associated with oseltamivir prescription by univariable analysis were having a younger age. None of the pre-specified high-risk conditions were associated with prescription, so a multivariable model could not be created. Oseltamivir use was significantly higher in patients admitted to the ICU (36·8% versus 18·6%) or that died (39·1% versus 18·3%), reflecting a tendency of higher prescription rates in those more critically ill.

We found that the use of oseltamivir in hospitalized patients was low and that only a younger age was associated with increased prescription rates. Reasons for low prescription rates may include a lack of perceived benefit of the drug to alter morbidity or mortality, as there has been a paucity of randomized trials on this subject. Other reasons may include the time delay of patients to present to the ER as well as the time delay inherent in laboratory testing, coupled with the misconception that treatment is only effective within the first 48 hours of illness, which was the time period cut-off for previous community-based studies and has been questioned in the inpatient setting. Finally, many influenza infections were likely incorrectly diagnosed as bacterial infections, suggested by the high rate of antibiotic prescription in this study. Our data illustrate the need to optimize the use of oseltamivir in those with influenza who are hospitalized.

Our findings reflect data from one large Canadian centre and may not reflect the prescribing practices in the wider population of inpatients in Canadian hospitals. In addition, the number of patients included in the study may have been too small to show associations between certain risk factors and oseltamivir use.
